# IL-33 drives the production of mouse regulatory T cells with enhanced in vivo suppressive activity in skin transplantation

**DOI:** 10.1111/ajt.16266

**Published:** 2020-09-15

**Authors:** Kento Kawai, Masateru Uchiyama, Joanna Hester, Fadi Issa

**Affiliations:** 1 Transplantation Research Immunology Group, Nuffield Department of Surgical Sciences, University of Oxford, Oxford, UK; 2 Department of Surgery, Teikyo University, Tokyo, Japan

**Keywords:** animal models: murine, basic (laboratory) research/science, cellular transplantation (non-islet), chemokines/chemokine receptors, cytokines/cytokine receptors, immunobiology, T cell biology

## Abstract

Regulatory T cells (Tregs) are crucial mediators of immune homeostasis with the ability to modulate allogeneic response and control transplant rejection. Although Treg-based cell therapies have shown immense promise, methods to optimize current strategies are critical for successful implementation within the clinic. IL-33 is a cytokine with pleiotropic properties and effects on Treg function and development. In this study, we explored the unique properties of Treg populations activated through the IL-33/ST2 pathway, aiming to exploit their tolerogenic properties for cell therapy. We show that treatment with exogenous IL-33 results in a generalized downregulation of genes critical to T cell biology together with an upregulation of Treg-associated genes. Tregs that develop in response to IL-33 upregulate critical Treg-associated markers, yet without developing enhanced in vitro suppressive capacity. Conversely, these Tregs display potent regulatory activity in vivo, promoting long-term skin allograft survival in a stringent transplantation model. Detailed transcriptomic and immunophenotypic analyses of IL-33–expanded Tregs reveal an enhancement in graft-homing chemokine receptors, which may be partly responsible for their superior in vivo activity that is not reflected in vitro. IL-33 treatment is therefore an attractive adjunctive strategy for patients receiving Treg cell therapeutics.

## 1 Introduction

CD4^+^ regulatory T cells (Tregs) are a distinct subpopulation of T cells with immune homeostatic activity specified by expression of the X-linked transcription factor FOXP3.^1^ A number of promising clinical trials have highlighted the potential for boosting Treg numbers to rebalance immune function for the treatment of autoimmune diseases, graft versus host disease, and solid organ transplantation.^2^ Among the current clinical trials, our group is a partner in the EU-funded ONE Study (Phase I)^3^ and Medical Research Council-funded TWO Study (Phase II) trials, which aim to investigate the safety and therapeutic efficacy of Tregs in living donor renal transplantation.

Currently, Treg cell therapy involves either the administration of ex vivo–expanded autologous Tregs^2,4–7^ or the in vivo expansion/activation of endogenous Tregs with low-dose IL-2 treatment.^8–11^ Both methods are limited by their ability to expand a pure population of functional Tregs to the high numbers required for therapy. IL-2 therapy is limited by its off-target effects that can result in the expansion of proinflammatory cells,^12,13^ whereas ex vivo Treg expansion can result in effector T cell overgrowth.^14^ Much work has been devoted in refining these 2 methods, specifically in manipulating molecular pathways to expand a greater number of Tregs and/or to increase their suppressive potency.

Modulation of the IL-33/ST2 pathway may be an attractive strategy for enhancing the efficacy of Treg-based cell therapy. Serum stimulation-2 (ST2 or IL1RL1) is a member of the IL-1 superfamily with only one described ligand: IL-33. IL-33 is thought to serve as an endogenous danger signal or alarmin in response to tissue damage or inflammation within epithelial and endothelial cells.^15,16^ ST2 is stably expressed on Th2 cells, mast cells, and group II innate lymphocytes.^17^ The IL-33/ST2 system has a role in the pathophysiology of a number of inflammatory conditions including allergy,^18^ atherosclerosis,^19^ and colitis.^20^

Recent mouse studies have described a role for IL-33 in Treg expansion with a positive feedback effect that results in upregulation of ST2 following IL-33 stimulation.^21,22^ These ST2^+^ Tregs localize predominantly in nonlymphoid tissue.^23^ There is evidence that ST2^+^/IL-33-responsive Tregs are potently anti-inflammatory^21,24–27^; however, the mechanisms whereby the IL-33/ST2 axis acts to enhance Treg function remain unclear, particularly as many of these mechanisms may be shared with Th1 and Th2 cells.^28–30^ IL-33 recruits both GATA-3 and RNA polymerase II to the *Foxp3* promoter and *Il1rl1* locus in a TGF-β–dependent manner.^28,31^ Through this positive feedback loop, IL-33 may indirectly upregulate Foxp3, while also enhancing ST2 expression. Moreover, other studies suggest that tissue-resident ST2^+^ Tregs depend on transcriptional regulators BATF and IRF4 for their differentiation.^23,32^

An important role exists for the IL-33/ST2 axis in the control of Treg function and development. Nonetheless, due to its pleiotropic nature and role in tissue-driven inflammation, harnessing its regulatory properties for therapeutic purposes is challenging. Thus to investigate the role of IL-33/ST2 in immune modulation as a route into enhancing Treg production techniques, we performed an indepth characterization of the effects of stimulation of the IL-33/ST2 axis on Tregs in vivo and assessed their ability to prevent transplant rejection.

## 2 Methods

### 2.1 Mice

CBA (CBA, H-2^k^), CBA Foxp3^GFP^ (H-2^k^), CBA Rag1^-/-^ (H-2^k^), and C57BL/6 (H-2^b^) were housed in the Biomedical Services Unit of the John Radcliffe Hospital (Oxford, UK). In the indicated experiments, mice were administered intraperitoneal (i.p.) injections with either phosphate-buffered saline (PBS) or recombinant IL-33 (1 μg/mouse/d, BioLegend) each day from days 1 to 6 and sacrificed for analysis on day 7. Animal care was conducted in accordance with the Animals (Scientific Procedures) Act 1986.

### 2.2 Flow cytometry

Single cell suspensions were prepared from spleens, peripheral blood, and peripheral lymph nodes (subiliac and axillary) and stained with the listed antibodies as per the manufacturer’s protocol. Cells were stained with 7-AAD (eBioscience, Thermo Fisher) to distinguish live from dead cells. The Foxp3/Transcription Factor Staining Buffer Set and the Fixation and Permeabilization kit (Invitrogen) were used for intracellular staining. Data were acquired with FACSCanto II (BD Biosciences) and analyzed with FlowJo software (Treestar). The following antibodies were used for flow cytometry (clone; manufacturer): CD3 (17A2; eBioscience), CD3 (145-2C11; eBioscience), CD4 (GK1.5; eBioscience), CD8 (53-6.7; eBioscience), Foxp3 (FJK-16s; eBioscience), CD25 (PC61; eBioscience), CD25 (PC61; BD Biosciences), ST2 (RMST2-2; eBioscience), CD62L (MEL-14; BD Biosciences), CD39, (24DMS1; eBioscience), CD73 (TY/11.8; eBioscience), CD44 (IM7; Biolegend), CD44 (IM7; eBioscience), CTLA-4 (UC10-4B9; eBioscience), CCR4 (2G12; eBioscience), CCR2 (SA203G11; Biolegend), CCR5 (7A4; eBioscience), CCR7 (4B12; Biolegend), PD-1 (RMP1-30; eBioscience), and ICOS (C398.4A; eBioscience). Absolute cell numbers were calculated by mixing a known number of counting beads (Invitrogen) with the cell sample before assaying by flow cytometry. Absolute cell number numbers were then calculated by comparing the ratio of bead events to cell events.

### 2.3 In vitro suppression assays

Splenocytes from CBA Foxp3^GFP^ (H-2^k^) mice treated with PBS or IL-33 (1 μg/mouse/d for 6 consecutive days) were processed into single cell suspensions. Cells were then enriched for CD4^+^ cells using the Dynabeads Mouse CD4 cells kit (Invitrogen, Thermo Fischer, cat 11415D) and subsequently isolated for CD25^+^ regulatory T cells (Treg) and CD25^neg^ effector T cells (Teff) cells using an anti-CD25 PE antibody (eBioscience, Thermo Fisher, clone PC61.5) and anti-PE Microbeads (Miltenyi Biotec Ltd., cat 130-048-801). CD4^+^CD25^+^ (Treg) and CD4^+^CD25^neg^ (Teff) cell numbers were counted using trypan blue staining, a hemocytometer, and microscope. All cell cultures were incubated at 37°C and 5% CO_2_ in complete medium (Sigma Aldrich) supplemented with penicillin/streptomycin (Sigma Aldrich), L-glutamine (Sigma Aldrich), and 10% FBS. For the bead-stimulated conditions, Teffs were first stained with violet proliferation dye 450 (BD), as per manufacturer’s instructions. Violet proliferation dye-labeled Teffs were then transferred to round-bottom 96-well plates at a concentration of 1 × 10^5^ cells/well with anti-CD3/anti-CD28 Dynabeads (Invitrogen, Thermo Fisher, cat 11456D) at a concentration of1 × 10^5^ cells/well. Control Tregs or IL-33-Tregs were then added at Treg:Teff ratios of 1:1, 1:2, 1:4, and 1:8. Cultures were incubated for 72-96 hours and harvested. Proliferation by Violet proliferation dye was measured using FACSCanto II and analyzed with FlowJo. Percentage of suppression was calculated using the following formula: (1-(div.index Treg + responders/div.index responders)) × 100. In allogeneic dendritic cell (DC)-stimulated conditions, allogeneic DCs were generated from C57BL/6 (H-2^k^) bone marrow that were cultured with GM-CSF and IL-4 (both 10 ng/mL, Peprotech) for 6 consecutive days in complete RPMI and then harvested. AlloDCs were added in in vitro suppression cultures at a concentration of 2 × 10^4^ cells/well with Teff and Tregs and incubated for a total of 114 hours with 3H thymidine (Perkin Elmer) being added in the last 18 hours. Cell proliferation data were measured in counts per minute (cpm) with a Betaplate reader. Percentage of suppression was calculated using the following formula: (1-(cpm Treg + responders/cpm responders)) × 100.

### 2.4 In vitro Treg expansion

Tregs were expanded in vitro with IL-33 stimulation using an adapted protocol, as described.^33^ CD4^+^CD25^+^ Treg populations from splenocytes of CBA Foxp3^GFP^ were isolated and stained with violet proliferation dye 450 (BD Biosciences). Bone marrow-derived allogeneic DCs from C57BL/6 mice were generated by preculturing with GM-CSF (10 ng/mL) and TGF-β (2 ng/mL, both Peprotech) for 6 days. CD4^+^CD25^+^ Tregs (1 × 10^5^/well) were cultured with allogeneic DCs (2 × 10^4^/well) with/without 10 ng/mL of rmIL-33 (Biolegend) for 14 days at 39°C. At day 7, half of the medium was replaced with new alloDC and/or rmIL-33 in the appropriate wells. Cultures were harvested for phenotypic analysis by flow cytometry.

### 2.5 Cell sorting for CD4^+^GFP^+^ Tregs

Splenocytes from CBA Foxp3^GFP^ (H-2^k^) mice treated with PBS or IL-33 were processed into single cell suspensions and first enriched for CD4^+^ cells using the Dynabeads Mouse CD4 cells kit (Invitrogen), as per the manufacturer’s instructions. CD4^+^ enriched cells were then stained with 7-AAD and anti-CD4 antibody for 30 minutes and washed with PBS. Cells were sorted for CD4^+^GFP^+^ and CD4^+^GFP^neg^ cells using the FACSAria II (BD Biosciences).

### 2.6 Skin transplantation

CBA Rag1^-/-^ (H-2^k^) were reconstituted intravenously with 2.5 × 10^4^ CD4^+^GFP^+^-sorted Tregs from either PBS- or IL-33-treated CBA Foxp3^GFP^ (H-2^k^) mice and/or 5 × 10^4^ CD4^+^GFP^neg^ Teffs from PBS-treated CBA Foxp3^GFP^ (H-2^k^) mice. In the subsequent day after cell transfer, mice were transplanted with full-thickness tail skin grafts under general anesthetic.^34^ Grafts were monitored for 100 days posttransplant and rejection was defined as complete destruction of the skin.

### 2.7 Nanostring gene expression

For transcriptomic analysis of splenocytes of PBS- or IL-33-treated CBA (H-2^k^) mice, 1 × 10^6^ whole splenocytes from each mouse were isolated. For analysis of CD4^+^GFP^+^ Tregs, all splenic Tregs sorted from each mouse were taken for RNA isolation. For analysis of skin grafts, 15 cryosections (20 μm thickness) for each skin graft were taken for isolation. RNA was isolated using RNeasy Micro kit (Qiagen) and analyzed with the nCounter Sprint Profiler using the Mouse PanCancer Immune Profiling panel and the nCounter Analysis System –with nSolver 4.0 software (all Nanostring).

### 2.8 Statistics

The statistical tests used for each experiment are stated in the corresponding figure legends. In experiments comparing 2 independent groups, unpaired 2-tailed Student’s *t* tests with Welch’s corrections were used. Allograft survival data were analyzed using the log-rank test. Statistics were considered significant at *P* < .05. All statistical analysis was performed using GraphPad Prism Software (GraphPad Software Inc), apart from Nanostring data, which were analyzed using nSolver 4.0 (Nanostring) using the basic and advanced modules.

### 2.9 Study approval

All mouse experiments were performed using protocols approved by the Committee on Animal Care and Ethical Review at the University of Oxford and in accordance with the UK Animals (Scientific Procedures) Act 1986 and under PPL number P8869535A.

## 3 Results

### 3.1 In vivo IL-33 treatment results in downregulation of integral T cell genes

**Figure 1:**
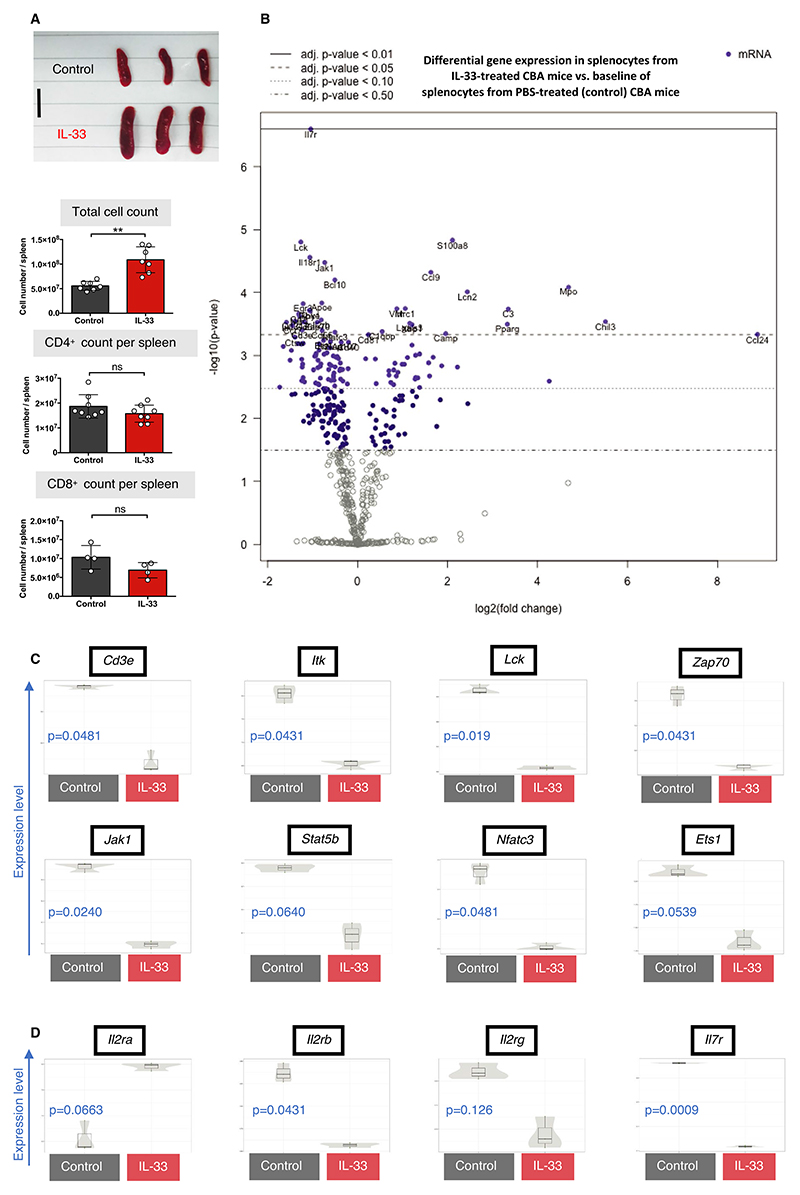
In vivo IL-33 treatment results in an overall downregulation of integral T cell–associated genes. CBA/Ca (H-2^k^) mice were injected with either PBS (control, black) or recombinant IL-33 (1 μg/d, red) for 6 consecutive days, and spleens were harvested 24 h after last injection and analyzed by flow cytometry. RNA from total splenocytes was isolated from PBS- (n = 3) or IL-33-treated (n = 3) H-2^k^ mice for gene expression analysis. A, Representative images (scale bar: 1 cm) and graphs of spleens from H-2^k^ PBS- or IL-33–treated mice with total splenocyte count and CD4^+^ (n = 8 per group) and CD8^+^ (n = 4 per group) counts per spleen (unpaired *t* test) (**P < .01; ns = not significant). B, Volcano plot revealing the most differentially expressed genes, relative to a baseline of control mice. C, Selected genes associated with T cell signaling and (D) cytokine receptor expression are represented in univariate scatter violin plots. B-D, Adjusted *P* value calculated with control of Benjamini-Yekutieli False Discovery Rate (FDR) (adjusted *P* > .05 considered significant, FDR thresholds indicated within volcano plot) [Color figure can be viewed at wileyonlinelibrary.com]

Because IL-33 acts both as an alarmin and Treg-promoting cytokine, we investigated the systemic effects of treatment with recombinant IL-33 in vivo. Previous studies have demonstrated that in vivo IL-33 treatment results in significant infiltration of eosinophils, myeloid cells, and plasma cells within the spleen.^15,35^ Consistent with these reports, after 6 consecutive days of IL-33 treatment (1 μg/d, harvested on day 7), we observed significant splenomegaly and a nearly 2-fold increase in splenocyte cell numbers in IL-33–treated mice compared with saline-treated control mice (*P* = .0012, Figure 1A). Despite this, there was no significant increase in total CD4^+^ or CD8^+^ cell counts upon IL-33 treatment, but there was an increase in the CD4 proportion within CD3 cells, together with a significant decrease of the CD8 proportion (Figure S1A). To establish a more comprehensive overview of the changes in immune cell composition with IL-33 treatment, we used multiplexed gene expression analysis. Relative to control mice, IL-33–treated mice had a substantial downregulation in the majority of immune *pathway scores*, calculated as the first principal component of each pathway’s normalized gene expression (Figure S1B). Notably, genes associated with adaptive immunity, cell cycle, chemokine and receptors, and cytokine and receptor pathway scores were significantly down-regulated. IL-33 treatment resulted in a differential expression of 35 genes across total leukocytes isolated from treated mice (adjusted *P* ≤ .05, Figure 1B and Table S1). Many of the most differentially downregulated genes were those coding transcription factors and receptors central to the T cell receptor (TCR) signaling pathway, including *Cd3e, Lck, Itk*, and *Zap70* (Figure 1C). We also observed an overall downregulation of genes such as *Jak1, Ets1, Stat5b*, and *Nfatc3* that encode regulators of major pathways within T cells, as well as genes such as *Lgals3, Xbp1, Pparg* (all upregulated), and *CD55* (downregulated), which have well-documented roles within T cell development and survival (Figure S1C). Collectively, IL-33 treatment resulted in an overall reduction in the number of transcripts associated with T cell biology. Because absolute numbers of T cells did not change significantly, this reduction in transcripts may be related to reduced T cell activity.

IL-33–treated mice also displayed a shift in gene expression for some of the major cytokine receptors. The most differentially down-regulated gene upon IL-33 treatment was *Il7r*, a molecule critical for T and B cell maintenance, but lowly expressed on Tregs.^36^ In addition, although there was a reduction in the IL-2 receptor (IL-2R) subunit genes *Il2rb* (CD122) and *Il2rg* (CD132), there was a selective increase in expression of *Il2ra* (CD25), which Tregs express preferentially and is important for their function (Figure 1D). This is further supported by the cell score analysis, which demonstrated a decreased relative abundance of proinflammatory cell types such as CD8^+^ T cells, NK cells, and Th1 cells, correlating with an increased relative abundance of Tregs (Figure S1D). Taken together, IL-33 treatment causes extensive transcriptomic changes that appear to promote an anti-inflammatory immune phenotype.

### 3.2 IL-33 modulates Foxp3 and ST2 expression dynamics

**Figure 2:**
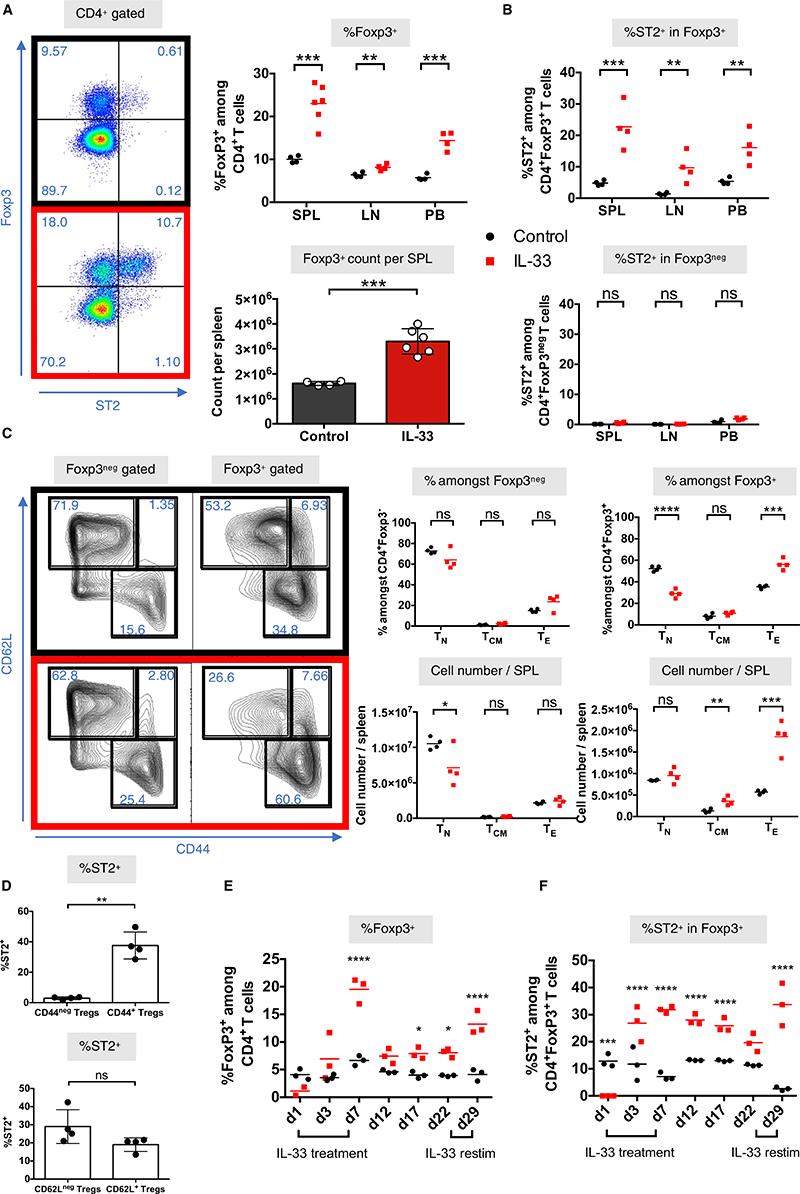
In vivo IL-33 treatment preferentially expands highly activated ST2^+^Foxp3^+^ Tregs. CBA/Ca (H-2^k^) mice were injected with either PBS (control, black) or recombinant IL-33 (1 μg/d, red) for 6 consecutive days and spleens (SPL), lymph nodes (LN), and peripheral blood (PB) were harvested 24 h after last injection and analyzed by flow cytometry. Representative dotplots (from splenocytes) and graphs of (A) Foxp3 and (B) ST2 expression within SPL, LN, and PB (unpaired *t* test, n = 3-6 mice). C, Representative dotplots (from splenocytes) and graphs of CD44 and CD62L expression and populations of naïve (T_N_, CD62L^+^CD44^neg^), central memory (T_CM_, CD62L^+^CD44^+^), and effector/effector memory T cells within Foxp3^+^ or Foxp3^neg^ populations. D, Graphs of the correlation of CD44 and CD62L expression with ST2 expression within Foxp3^+^ Treg populations (unpaired *t* test, n = 4). Peripheral blood time-course analysis of (E) Foxp3 and (F) ST2 expression from PBS or IL-33-injected H-2^k^. Injections were given on days 1 to 6 and days 23 to 28 (unpaired *t* test, n = 3) (**P < .01; ***P < .001; ****P < .0001; ns = not significant) [Color figure can be viewed at wileyonlinelibrary.com]

We next examined the effects of IL-33 treatment on CD4^+^ T cell subpopulations, finding a significant systemic expansion of CD4^+^Foxp3^+^ Tregs within the spleen, lymph nodes, and peripheral blood (Figure 2A). ST2 was almost exclusively expressed on Foxp3^+^ Tregs (Figure 2B). IL-33 treatment also resulted in a shift in CD4^+^Foxp3^+^ Tregs toward an effector phenotype with increased CD44 expression and reduced CD62L expression (Figure 2C). ST2 expression was largely limited to CD44^+^Foxp3^+^ cells, making it a surrogate marker for Treg activation (Figure 2D). Using an adapted protocol (Figure S2A),^33^ we also confirmed that Foxp3^+^ Tregs with high ST2 expression can be expanded significantly in vitro with IL-33 treatment and stimulation using tolerogenic DCs (bone marrow–derived C57BL/6 DCs precultured with GM-CSF and TGF-β, Figure S2B). With this protocol, IL-33 stimulation increased the proliferation of Foxp3^+^ populations (Figure S2C), with a similar shift toward an effector phenotype (CD44^+^CD62L^lo^, Figure S2D).

To assess how IL-33 treatment affects Foxp3/ST2 expression dynamics in vivo over time, we sampled the peripheral blood of control and IL-33-treated mice at multiple points during and after cessation of treatment (Figure 2E). We found that Foxp3 and ST2 expression peaked the day after treatment was stopped (day 7) and was followed by a drop in Foxp3 expression soon after (day 12), but with levels remaining significantly above control even 2 weeks after treatment. The drop in Foxp3^+^ Tregs within the peripheral blood may suggest their migration elsewhere or their requirement for continual IL-33 stimulation for survival. When IL-33 was readministered (days 23-28), we found that mice remained responsive to IL-33, with Foxp3 (Figure 2E) and ST2 (Figure 2F) levels upregulated to levels comparable with the primary treatment. The persistent effect on Foxp3 and ST2 levels within the peripheral blood even after IL-33 treatment was discontinued suggests that ST2^+^ Tregs remain able to survive peripherally, despite being most commonly located within tissues.^23,32,37,38^

### 3.3 IL-33 Tregs display enhanced suppressive activity in vivo but not in vitro

**Figure 3:**
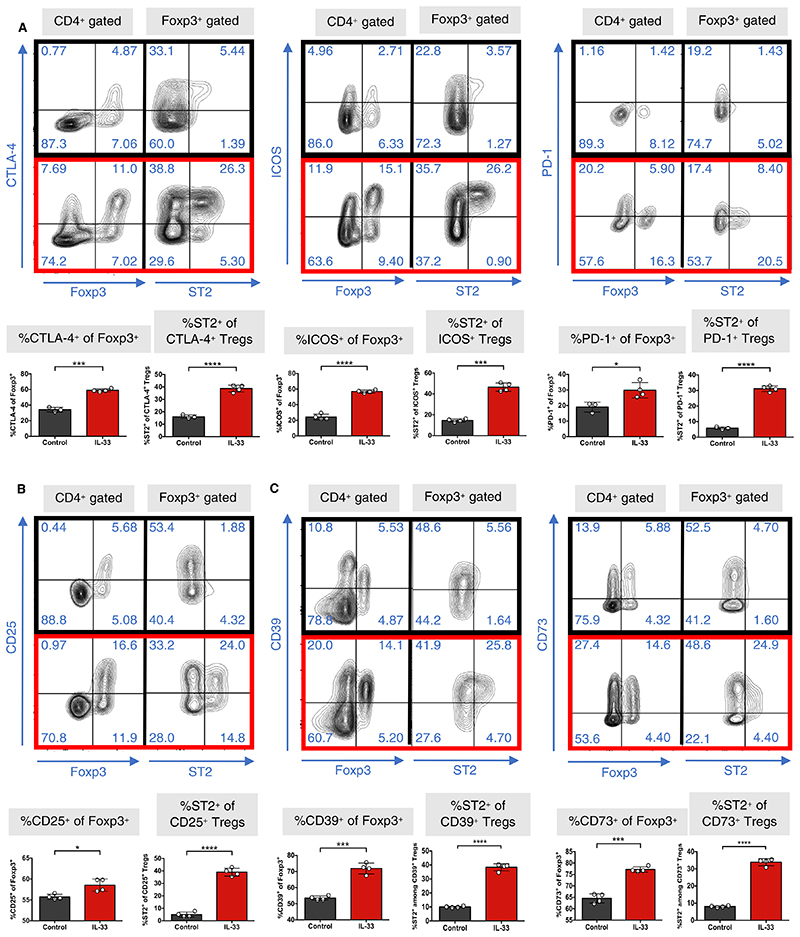
IL-33-Tregs upregulate Treg-specific functional molecules. CBA/Ca (H-2^k^) mice were injected with either PBS (control, black) or recombinant IL-33 (1 μg/d, red) for 6 consecutive days, and spleens were harvested 24 h after last injection and analyzed by flow cytometry. Representative dotplots and graphs of expression of (A) the costimulatory markers CTLA-4, ICOS, and PD-1; (B) CD25; and (C) the ectonucleotidases CD39 and CD73, all within Foxp3^+^ populations and expression of ST2 within their respective Treg populations (unpaired *t* test, n = 4) (*P < .05; ***P < .001; ****P < .0001; ns = not significant) [Color figure can be viewed at wileyonlinelibrary.com]

To explore the phenotype of IL-33-expanded Tregs (IL-33 Tregs), we measured their expression of markers associated with Treg-mediated suppression. IL-33 Tregs significantly upregulated their expression of the costimulatory/coinhibitory molecules CTLA-4, ICOS, and PD-1 (Figure 3A). The ectonucleotidase molecules CD39 and CD73 were also upregulated (Figure 3B), suggesting enhanced activity through the generation of adenosine^39–41^ and/or cAMP transfer.^42,43^ CD25 expression levels were also increased (Figure 3C), correlating with transcriptomic data (Figure 1D). Finally, ST2 expression within each of the respective Foxp3^+^ populations was significantly higher (Figure 3A-C).

**Figure 4:**
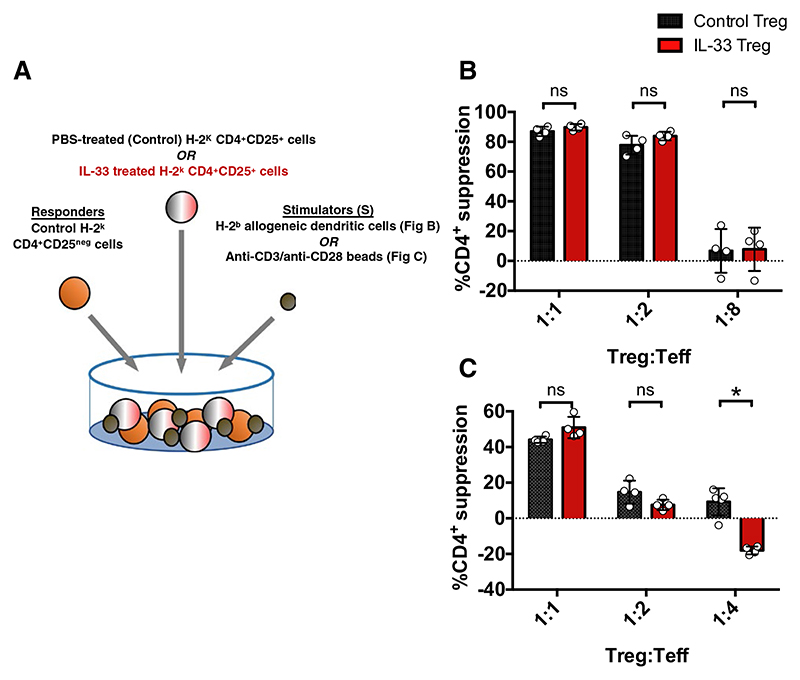
IL-33-Tregs do not demonstrate suppressive advantage in vitro. A, Schematic of in vitro suppression assay design. PBS- or IL-33-treated CBA/Ca splenocyte-derived CD4^+^CD25^+^ Tregs were cultured with CD4^+^CD25^neg^ Teff responders stimulated with (B) H-2^b^ allogeneic DC cells or (C) anti-CD3/anti-CD28 beads to assess their suppressive potency in vitro. B,C, Data are shown as means ± SD. Unpaired, independent groups (1:1, 1:2, 1:4, and 1:8) of 2 were analyzed using unpaired *t* tests (*P < .05; ns = not significant) [Color figure can be viewed at wileyonlinelibrary.com]

We next investigated whether these molecular changes resulted in enhanced functional activity in vitro. Data assessing the functional suppression of IL-33-stimulated Tregs in vitro from previous studies are conflicting, ranging from data showing enhanced, reduced, or no difference in suppression relative to non-IL-33-stimulated Tregs.^22,26,33,44^ To further explore this issue, we assessed the capacity of IL-33 Tregs to suppress either allogeneic DC-driven or polyclonal bead-driven responder proliferation (Figure 4A). Of interest, despite the favorable molecular changes, we found no functional advantage in suppression for IL-33 Tregs compared with control Tregs under either condition (Figure 4B,C). At lower ratios, however, we found that IL-33 Tregs were significantly less suppressive relative to control Tregs in the bead-stimulation assay. However, the significance of this finding is unclear as it was not reflected in the allogeneic-DC–stimulated assay.

**Figure 5:**
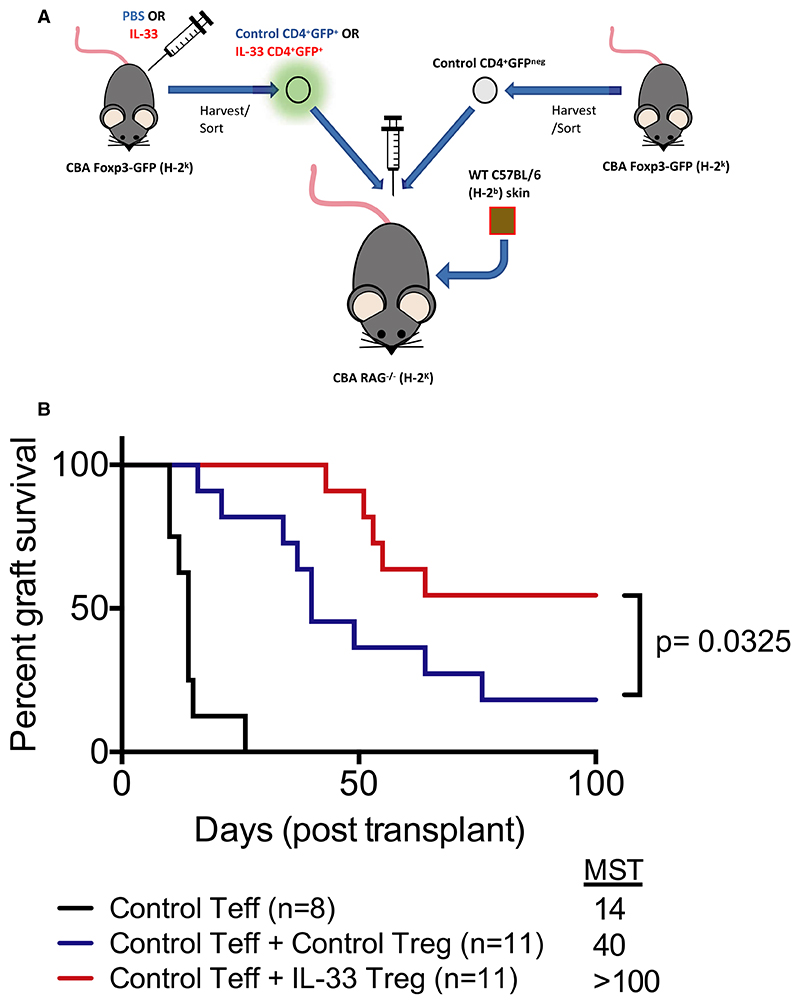
IL-33 Tregs are potently functional in vivo. A, Schematic of experimental skin transplant model design. CBA/Ca Rag1^-/-^ (H-2^k^) received control CD4^+^GFP^neg^ effector T cells (Teff) with or without CD4^+^GFP^+^ Tregs from H-2^k^ Foxp3-GFP mice that were treated with PBS or IL-33 (1 μg/d for 6 consecutive days and sacrificed on day 7). One day later, mice received an allogeneic fully MHC mismatched H-2^b^ skin allograft, which was monitored for rejection for 100 days posttransplant. B, Graft survival graph (2 independent assays) of 3 groups of mice receiving wildtype (WT) Teffs only (n = 8, black), WT Teffs + control Tregs (n = 11, blue), and WT Teffs + IL-33-Tregs (n = 11, red). Survival data were analyzed using log-rank test (*P* > .05 considered significant) [Color figure can be viewed at wileyonlinelibrary.com]

Nonetheless, in vitro assays cannot fully recapitulate the full spectrum of elements that may be relevant to Treg activity. Previous studies have demonstrated that exogenous IL-33 treatment can prolong minor antigen-mismatched heart and skin allograft survival.^22,45–47^ However, no studies have assessed the in vivo function of Tregs isolated from IL-33–treated mice within the context of solid organ transplantation. To address this, we used a well-characterized skin transplantation model in which H-2^k^ RAG^-/-^ mice receive a fully MHC-mismatched H-2^b^ skin allograft and an adoptive transfer of H-2^k^ effector CD4^+^ cells that results in graft rejection with a median survival time (MST) of 14 days. We treated these mice with Tregs at ratios that are known to extend graft survival only moderately (Figure 5A). As expected, treatment with control Tregs resulted in a modest extension of allograft survival (MST 40 days, Figure 5B, Table S2). Remarkably, IL-33-Treg treatment resulted in long-term engraftment in this stringent model (MST >100 days, *P* = .036). In mice that had achieved long-term (<100 days) survival, analysis of the spleen, lymph nodes, and peripheral blood revealed no significant differences in the proportion of Foxp3^+^ cells within the CD4^+^ population between control Treg and IL-33-Treg treated groups (Figure S3A). However, these data are limited by the low number of mice that achieved long-term survival in the control group. Nonetheless, analysis of Foxp3^+^ Tregs in the IL-33-Treg group demonstrated a higher expression of ST2 within all organs, suggesting long-term survival of these adoptively transferred cells (Figure S3B). Taken together, our findings reveal an enhanced graft-protective effect of IL-33-Tregs in vivo.

### 3.4 IL-33 Tregs upregulate genes critical for suppression and migration of Tregs to allograft

**Figure 6:**
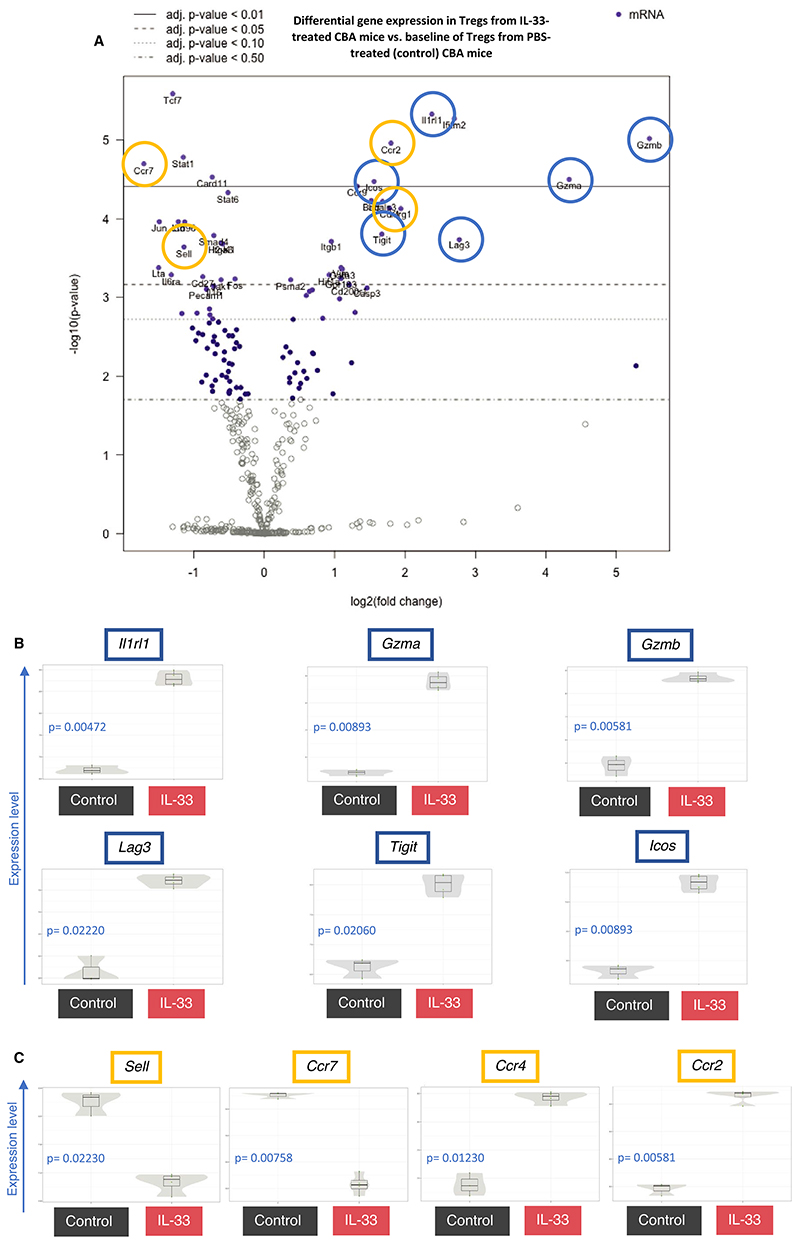
IL-33-Tregs upregulate genes critical for suppression and migration of Tregs to tissue. CBA/Ca (H-2^k^) mice were injected with either PBS (control, black) or recombinant IL-33 (1 μg/d, red) for 6 consecutive days, and spleens were harvested 24 h after last injection. RNA from splenocyte-derived CD4^+^CD25^+^ Tregs were FACS-sorted from PBS- (n = 3) or IL-33-treated (n = 4) H-2^k^ mice for gene expression analysis. (A) Volcano plot reveals the most differentially expressed genes, relative to a baseline of control mice. Genes associated with Treg suppressive function (B, blue) and genes associated with Treg homing (C, green) are represented in scatter violin plots. Adjusted *P* value calculated with control of Benjamini-Yekutieli False Discovery Rate (FDR) (adjusted *P* > .05 considered significant, FDR thresholds indicated within volcano plot) [Color figure can be viewed at wileyonlinelibrary.com]

We next sought to understand the features that underlie this in vivo functional advantage. Tregs were flow-sorted from IL-33-treated and control mice and assessed by multiplexed quantitative transcriptomic analysis (Figure 6A and Table S3). We next flow-sorted and analyzed the transcriptomic changes in CD4^+^CD25^+^ Tregs. By selecting cells expressing CD25, we removed any confounding of an increase in the proportion of activated Tregs after IL-33 treatment. It is notable that the majority of CD25^+^ cells also co-expressed Foxp3 in both groups (Figure 3B). As expected, 2 of the most differentially regulated genes were signature genes associated with IL-33 Tregs, *Il1rl1* and *Gata3* (Figure 6B and Figure S4A). In addition, *Tcf7* (downregulated), *Batf, Cd200r1*, and *Klgr1* (all upregulated) were also among the most differentially expressed genes, which have recently been identified as signature genes within ST2^+^ tissue-resident Tregs.^23^ Consistent with the immunophenotyping data, many of the most differentially up-regulated genes were those commonly associated with Treg suppressive function such as *Tigit*, *Icos*, and *Lag3*. Two of the most upregulated genes in IL-33 Tregs were the granzyme genes *Gzma* and *Gzmb*, both molecules are important for Treg activity.^48^ Of interest, granzyme B is known to cleave IL-33 into more mature forms and thus may indicate activation of another mechanism by which granzymes may be involved in IL-33–dependent activation signaling.^49,50^

**Figure 7:**
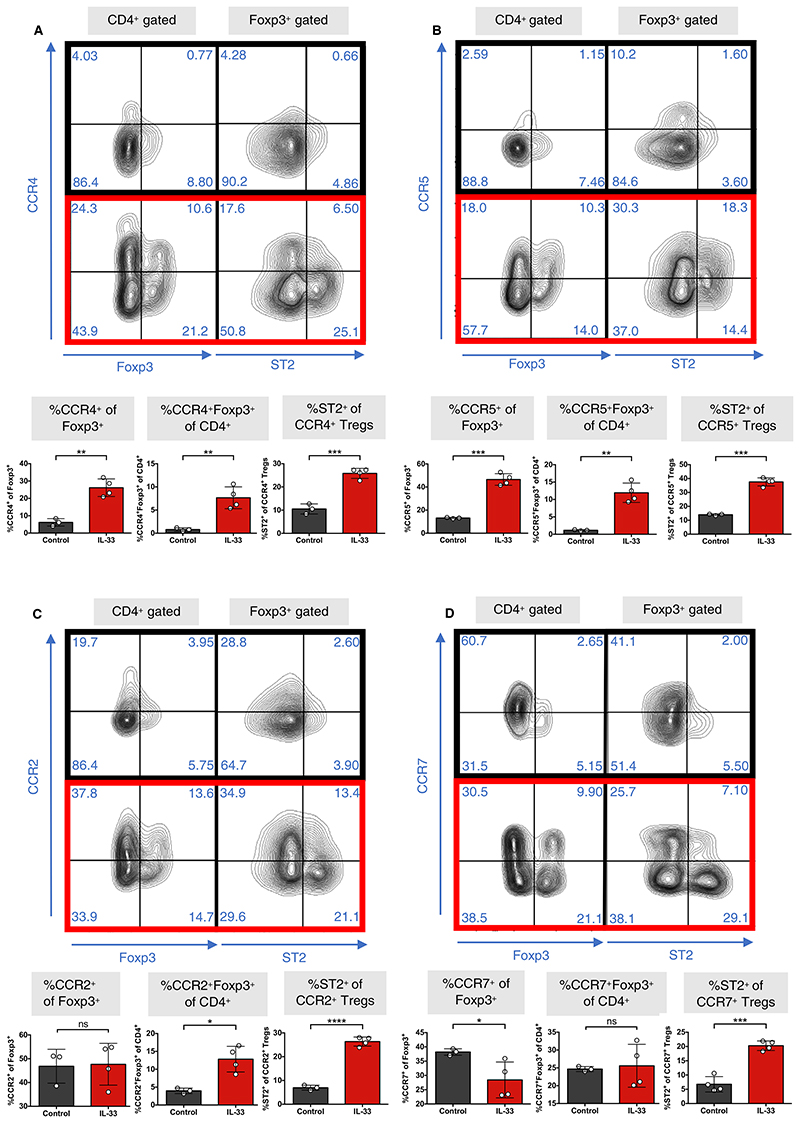
IL-33-Tregs express specific patterns of graft and lymph node homing chemokine receptor molecules. CBA/Ca (H-2^k^) mice were injected with either PBS (control, black) or recombinant IL-33 (1 μg/d, red) for 6 consecutive days, and spleens (SPL) were harvested 24 h after last injection. A, Representative dotplots show CCR4 vs Foxp3 in CD4^+^ gated populations and CCR4 vs ST2 expression in Foxp3^+^ gated populations. Graphs depict percentage of CCR4^+^ of Foxp3^+^ populations, percentage of ST2^+^ of CCR4^+^ Tregs, and percentage of CCR4^+^Foxp3^+^ of CD4^+^ cells within Tregs. Data are also shown for (B) CCR5, (C) CCR2, and (D) CCR7 (unpaired *t* test, n = 4) (*P < .05; **P < .01; ***P < .001; ****P < .0001; ns = not significant) [Color figure can be viewed at wileyonlinelibrary.com]

Examination of chemokine receptor genes revealed a highly significant differential upregulation of *Ccr2* and *Ccr4* (Figure 6C). CCR2 and CCR4 are both critical to the migration of Tregs to the allograft,^51–54^ and are also associated with enhanced regulatory function.^55,56^ Two of the most differentially downregulated genes in IL-33 Tregs were *Sell* and *Ccr7*, both of which are strongly associated with Treg homing to the lymph node.^51,57,58^ This was consistent with the downregulation of CD62L upon IL-33 treatment (Figure S4B). Moreover, the lymphotoxin genes *Lta* and *Ltb*, which guide Tregs from the allograft to the lymph node via afferent lymphatics,^59^ were also 2 of the most differentially downregulated genes (Figure S4C). Changes in chemokine transcripts were confirmed by flow cytometry for cell surface protein expression (Figure 7A-C; Figure S4D). To explore local immune infiltration within the allografts in both groups, we performed Nanostring transcriptomic analysis of rejecting skin grafts from the control Treg group (n = 2) and grafts that reached long-term survival from the IL-33-Tregs group (n = 2). We found that *Il2ra, Ctla4*, and *Il1r2* were among the most significantly upregulated genes in regulated skin grafts from the IL-33-Treg group relative to those of the control Treg groups (Figure S5), suggesting enhanced Treg infiltration.

Collectively, our findings suggest that IL-33 Tregs have functional immune regulatory advantages in vivo that are not apparent in vitro. This disparity may be related to functional changes that cannot be measured in vitro, such as enhanced allograft homing, although other mechanisms such as enhanced survival may also be active and require further investigation.

## 4 Discussion

An understanding of how Tregs and other immune cell populations are modulated in response to IL-33 has previously been difficult to characterize in vivo. In this study, we present an in-depth characterization of the function and phenotype of Tregs that develop after IL-33 treatment. Our findings demonstrate the development of a generalized immunosuppressive state after IL-33 treatment that results in the production of a Treg population with upregulated graft-homing molecules. The treatment of mice with Tregs that express these grafthoming molecules at the point of transfer may facilitate their rapid migration to the transplant to suppress early alloresponses.

Recent human data point toward the potential translatability of these findings, demonstrating increased proportion or abundance of ST2^+^ Tregs within the peripheral blood and colon of colorectal cancer patients,^60^ and a role for IL-33 in the expansion of IL-13-secreting Tregs.^25^ More recently, heart allografts from transplant patients have been shown to have enhanced release of IL-33, suggesting a possible tissue-autonomous mechanism to control graft rejection.^61^ In contrast, findings from Lam et al noted very minimal ST2 expression in Tregs derived from various human tissues,^62^ which may suggest that ST2 in human Tregs is not commonly detected or easily expressed in the quiescent state. Overall, these findings invite further exploration of the role of the IL-33/ST2 pathway in Tregs in pathological states. Understanding the clinical relevance in humans will require the development of human recombinant IL-33 proteins and specific ST2 antibodies that can target this pathway for translational purposes.

Although the mechanisms by which specific Treg populations exert their suppressive functions are highly variable, expression of certain functional molecules provides an indication of their preferred mechanisms. In our analysis, we sought to measure the percentage of Tregs expressing conventional suppressive markers that may also express ST2, as we did not directly compare ST2^+^ vs ST2^neg^ Tregs in our functional assays. Our findings are in agreement with previous studies that have observed an upregulation of costimulatory markers (CTLA-4, ICOS, and PD-1) and an enhanced expression of CD25 after IL-33 treatment.^33^ The upregulation in the ectoenzymes CD39/CD73 and their positive correlation with ST2 expression within Tregs may form part of a larger facet to IL-33-Treg biology. These molecules augment the ability of IL-33 Tregs to inhibit Teff responses through the production of adenosine from ATP. Extracellular ATP can act as a danger signal in response to cell damage,^63^ with reports suggesting that IL-33 and ATP may act to induce the release of one another under inflammatory conditions.^64,65^ Thus coupled with reports that there is substantial release of IL-33, ATP, and other alarmin/DAMPS after transplantation, IL-33 Tregs may have a unique potential in modulating inflammation within an allograft microenvironment.^66–68^ This feature may also be related to the previously described protective functions of ST2^+^ Tregs in enhancing tissue repair in inflammatory environments through their upregulation of amphiregulin.^26,62,69^ Moreover, our analysis reveals that IL-33 Tregs may have a transcriptomic signature similar to tissue-resident Tregs, further supporting their role in modulating local inflammation.^23,32,38^ The role of this module of functional molecules in IL-33-Treg-mediated graft-protection therefore warrants further assessment.

In vitro data examining IL-33 in Treg biology have revealed differences that may be secondary to specific elements of the experimental design. These may be related to comparisons between ST2^+^ vs ST2^neg^ Tregs,^22,24,33^ pre-IL-33 stimulation^22,26,33^ vs incorporation of IL-33 directly within the culture,^26,44^ anti-CD3/anti-CD28 bead^22,33,44^ vs APC-stimulated conditions,^24^ and strain differences between C57BL/6 ^22,24,26,33^ and BALB/C mice.^44^ In an effort to understand how IL-33 affects overall immune balance, we sought to assess the suppressive capacity of the total Treg population that develops after IL-33 treatment, instead of focusing specifically on ST2^+^ vs ST2^neg^ Tregs. Our in vitro results underscore the importance of assessing cellular therapies in vivo, where whole animal-related mechanisms of function and migration may otherwise be lost.

The pleiotropic nature of IL-33 raises some concerns related to the induction of proinflammatory responses.^30,70,71^ However, the generalized T cell–downregulated state highlighted by our study of the entire leukocyte population transcriptome is reassuring. Previous studies have highlighted the therapeutic benefits of IL-33 treatment in transplantation.^22,45–47^ In these studies, protection was mediated through mechanisms that include the induction of Tregs, myeloid-derived suppressive cells, and Th2-skewing. Our adoptive transfer assays emphasize the integral role that Tregs have in isolation after IL-33 treatment. However, these assays are limited by an inability to precisely identify the timing of migration of Tregs to the site of the alloresponse. Previous work indicates that these cell migration molecules are important for graft infiltration^51,52,54,72^ and suppressive function.^55,56^ Notably, Zhang et al previously demonstrated that adoptively transferred *Ccr2^−/−^*, *Ccr4^−/−^*, or *Ccr5^−/−^* Tregs had significantly impaired allograft homing capacity, resulting in diminished graft survival in mice.^51^ Although studies have suggested that lymph node homing capability marked by the upregulation of molecules such as CCR7 and CD62L is also important for full graft-protective function of Tregs,^51,73,74^ we did not detect a significant difference in Treg proportions in graft-draining lymph nodes between mice treated with IL-33–induced Tregs or control Tregs when examined at the point of long-term survival (Figure S3).

Overall, our study characterizes the features by which in vivo IL-33 treatment results in an immune regulatory state, together with an indepth assessment of the effects this has on Treg populations. With low dose and mutein IL-2 therapy currently advancing through clinical trials as a method for in vivo Treg expansion in autoimmune diseases and transplantation,^13,75,76^ there is now an attractive argument for exploring IL-33 therapy for the same indications. Our study highlights an important advantage that IL-33 holds in promoting a generalized regulatory state while suppressing overall T cell pathways. This is of particular importance given emerging data suggesting proliferation of proinflammatory cell types after low-dose IL-2 treatment, and provides an alternative, focused, strategy for preferential Treg expansion.

## Supplementary Material

Supplementary Material

## Data Availability

The data that support the findings of this study are available from the corresponding author upon reasonable request.

## Abbreviations

DC: dendritic cell
IL-33: interleukin 33
IL-33 Treg: interleukin 33-expanded Treg
LN: lymph node
MST: median survival time
PB: peripheral blood
SPL: spleen
ST2: serum stimulation-2
TCR: T cell receptor
Teff: effector T cell
Treg: regulatory T cell
WT: wild-type
