# Supplementary material for: IL-33 drives the production of mouse regulatory T cells with enhanced in vivo suppressive activity in skin transplantation
Source: Am J Transplant. Author manuscript; Available in PMC 2022 Jul 23. (PMC7613121; doi:10.1111/ajt.16266)
Supplement: Supplementary Material [file EMS150705-supplement-Supplementary_Material.pdf]

	probe.ID	Log2 fold change	BY.p.value
**Il7r-mRNA**	NM_008372.3:1020	-1.04	0.000909
**S100a8-mRNA**	NM_013650.2:227	2.11	0.019
**Lck-mRNA**	NM_010693.2:1180	-1.26	0.019
**Il18r1-mRNA**	NM_001161842.1:620	-1.06	0.024
**Jak1-mRNA**	NM_146145.2:4080	-0.73	0.024
**Ccl9-mRNA**	NM_011338.2:1125	1.63	0.0287
**Bcl10-mRNA**	NM_009740.1:1168	-0.504	0.0325
**Mpo-mRNA**	NM_010824.2:1648	4.69	0.037
**Lcn2-mRNA**	NM_008491.1:190	2.44	0.0391
**Apoe-mRNA**	NM_009696.3:129	-0.795	0.0431
**Egr3-mRNA**	NM_018781.2:518	-1.21	0.0431
**Mrc1-mRNA**	NM_008625.1:3992	1.06	0.0431
**Vim-mRNA**	NM_011701.4:34	0.873	0.0431
**C3-mRNA**	NM_009778.2:285	3.35	0.0431
**Ikbke-mRNA**	NM_019777.3:618	-1.07	0.0431
**Thy1-mRNA**	NM_009382.3:425	-1.05	0.0431
**Cd7-mRNA**	NM_009854.1:234	-1.31	0.0431
**Ncr1-mRNA**	NM_010746.3:391	-1.29	0.0431
**Il2rb-mRNA**	NM_008368.3:2365	-0.807	0.0431
**Cd247-mRNA**	NM_001113391.2:215	-1.39	0.0431
**Chil3-mRNA**	NM_009892.2:823	5.51	0.0431
**Cd6-mRNA**	NM_001037801.2:1315	-1.18	0.0431
**Itk-mRNA**	NM_010583.3:404	-1.58	0.0431
**Zap70-mRNA**	NM_009539.2:1030	-0.894	0.0431
**Lgals3-mRNA**	NM_001145953.1:665	1.16	0.0431
**Pparg-mRNA**	NM_011146.1:1060	3.33	0.0431
**Xbp1-mRNA**	NM_013842.2:825	1.21	0.0431
**Ccr7-mRNA**	NM_007719.2:755	-1.44	0.0431
**Cd3e-mRNA**	NM_007648.4:380	-1.23	0.0481
**Cd55-mRNA**	NM_010016.2:1058	-0.787	0.0481
**C1qbp-mRNA**	NM_007573.2:630	0.548	0.0481
**Nfatc3-mRNA**	NM_010901.2:2260	-0.502	0.0481
**Camp-mRNA**	NM_009921.2:355	1.96	0.0481
**Ccl24-mRNA**	NM_019577.4:335	8.89	0.0481
**Cd81-mRNA**	NM_133655.2:575	0.243	0.0481

Supplementary Table 1. Significantly differentiated genes between splenocytes of PBS and IL-33-treated mice. Statistically significant (adjusted *p* value <0.05) differentially regulated genes between splenocytes of PBS (n=3) and IL-33-treated (n=3) mice (1μg/day for 6 consecutive days and sacrificed on day 7) are shown with respective Log2 fold change and probe ID. Adjusted *p* value calculated with control of False Discovery Rate (FDR) using Benjamini-Yekutieli method.

Condition	n	Survival (days)	MST
WT Teff only	8	10,10,12,14,14,14,15,26	14
WT Teff + Control Treg	11	16,21,34,37,40,40,49,64,76,100,100	40
WT Teff + IL-33-Treg	11	43,51,53,55,64,100,100,100,100,100,100	>100

Supplementary Table 2. IL-33 Tregs promote long-term skin allograft survival. Graft survival (days) of mice receiving WT Teffs only (n=8), WT Teffs + control Tregs (n=11), and WT Teffs + IL-33-Tregs (n=11).

	probe.ID	Log2 fold change	BY.p.value
Tcf7-mRNA	NM_009331.3:1810	-1.3	0.00472
Il1rl1-mRNA	NM_001025602.2:815	2.38	0.00472
Ifitm2-mRNA	NM_030694.1:87	2.7	0.00472
Gzmb-mRNA	NM_013542.2:1020	5.47	0.00581
Ccr2-mRNA	NM_009915.2:2965	1.8	0.00581
Stat1-mRNA	NM_009283.3:1590	-1.15	0.0073
Ccr7-mRNA	NM_007719.2:755	-1.71	0.00758
Card11-mRNA	NM_175362.2:545	-0.739	0.00893
Gzma-mRNA	NM_010370.2:188	4.33	0.00893
Icos-mRNA	NM_017480.1:142	1.56	0.00893
Ccr9-mRNA	NM_009913.6:820	1.32	0.00933
Stat6-mRNA	NM_009284.2:3465	-0.515	0.0103
Batf-mRNA	NM_016767.2:750	1.52	0.0114
Lgals3-mRNA	NM_001145953.1:665	1.68	0.0114
Ccr4-mRNA	NM_009916.2:394	1.78	0.0123
Klrg1-mRNA	NM_016970.1:825	1.94	0.0123
Ltb-mRNA	NM_008518.2:163	-1.22	0.0154
Jun-mRNA	NM_010591.2:2212	-1.49	0.0154
Cd96-mRNA	NM_032465.2:34	-1.13	0.0154
Tigit-mRNA	NM_001146325.1:730	1.67	0.0206
Smad4-mRNA	NM_008540.2:2885	-0.716	0.0206
Lag3-mRNA	NM_008479.1:1700	2.77	0.0222
Itgb1-mRNA	NM_010578.1:1855	0.953	0.0223
H2-K1-mRNA	NM_001001892.2:1370	-0.604	0.0223
Itga6-mRNA	NM_008397.3:910	-0.591	0.0223
Sell-mRNA	NM_001164059.1:664	-1.14	0.0233
Vim-mRNA	NM_011701.4:34	1.09	0.0397
Lta-mRNA	NM_010735.1:1115	-1.5	0.0397
Gata3-mRNA	NM_008091.3:1943	1.11	0.04
Il6ra-mRNA	NM_010559.2:2825	-1.32	0.0441
Hif1a-mRNA	NM_010431.2:1294	0.921	0.0441
Cd27-mRNA	NM_001033126.2:235	-0.873	0.0441
Gpr183-mRNA	NM_183031.2:238	1.09	0.0441
Fos-mRNA	NM_010234.2:1330	-0.415	0.0441
Jak1-mRNA	NM_146145.2:4080	-0.613	0.0441
Psma2-mRNA	NM_008944.2:136	0.376	0.0441
Cd200r1-mRNA	NM_021325.3:1090	1.21	0.0494

Supplementary Table 3. Significantly differentiated genes between control and IL-33-Tregs. Statistically significant (adjusted *p* value <0.05) differentially regulated genes between $CD4^{+}CD25^{+}$ Tregs of PBS (n=3) and IL-33-treated (n=4) mice, as described previously, are shown with respective Log2 fold change and probe ID. Adjusted *p* value calculated with control of False Discovery Rate (FDR) using Benjamini-Yekutieli method.

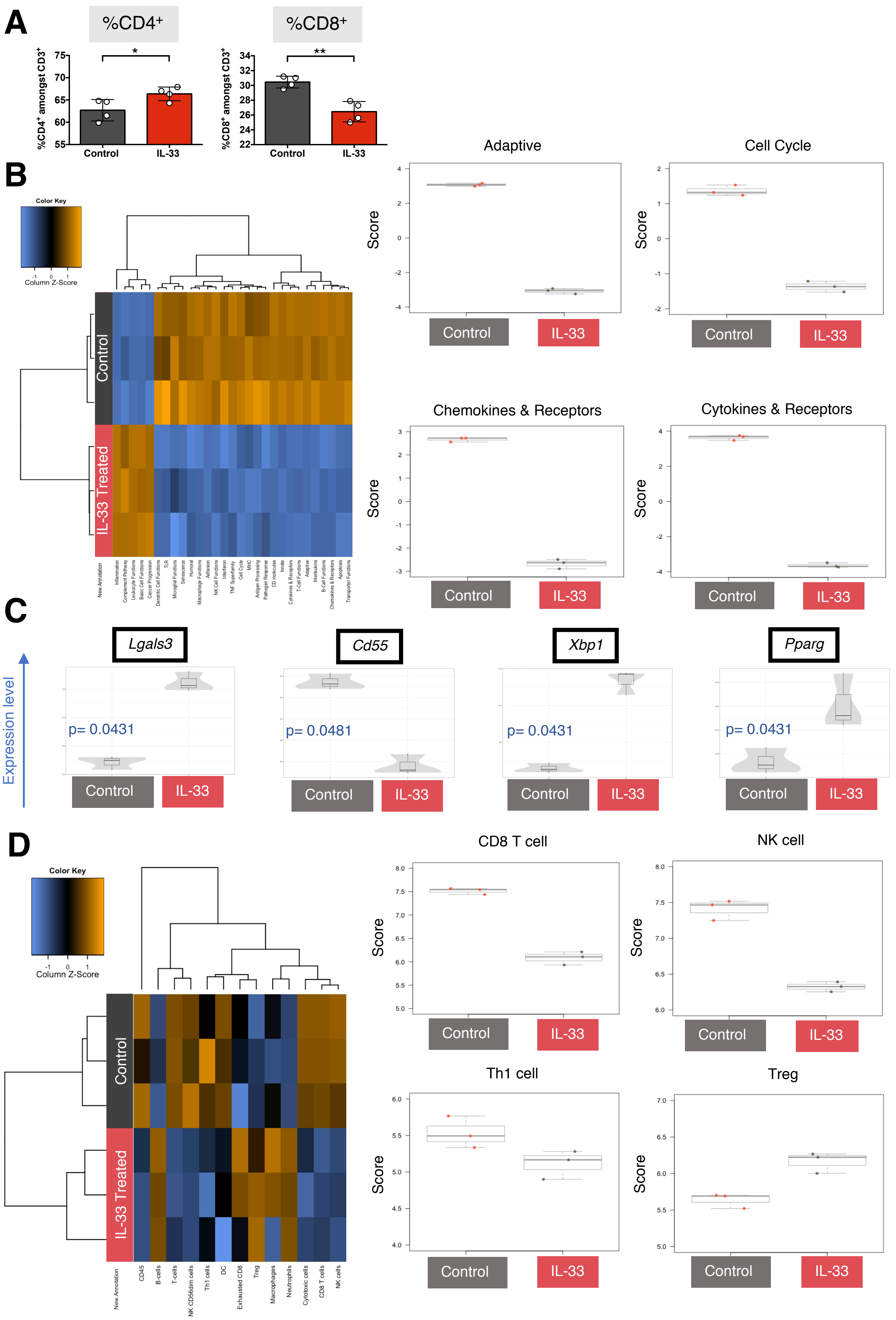

Supplementary Figure 1. CBA/Ca (H-2ᵏ) mice were injected with either PBS (control, black) or recombinant IL-33 (1ug/day, red) for 6 consecutive days, and spleens were harvested 24 hours after last injection and analyzed for flow cytometric and transcriptomic analysis. **(A)** Graphs of CD4 and CD8 expression. **(B)** Heat map and dot plots of gene expression profiles of pathway scores, which are fit using the first principal component of each sample based on the individual gene expression levels for all the measured genes within a specific pathway. **(C)** Select genes associated with T cell development and function are represented in scatter violin plots. **(D)** Heat map and dotplots of gene expression profiles of cell type scores, which are displayed on the same scale via a Z-transformation.

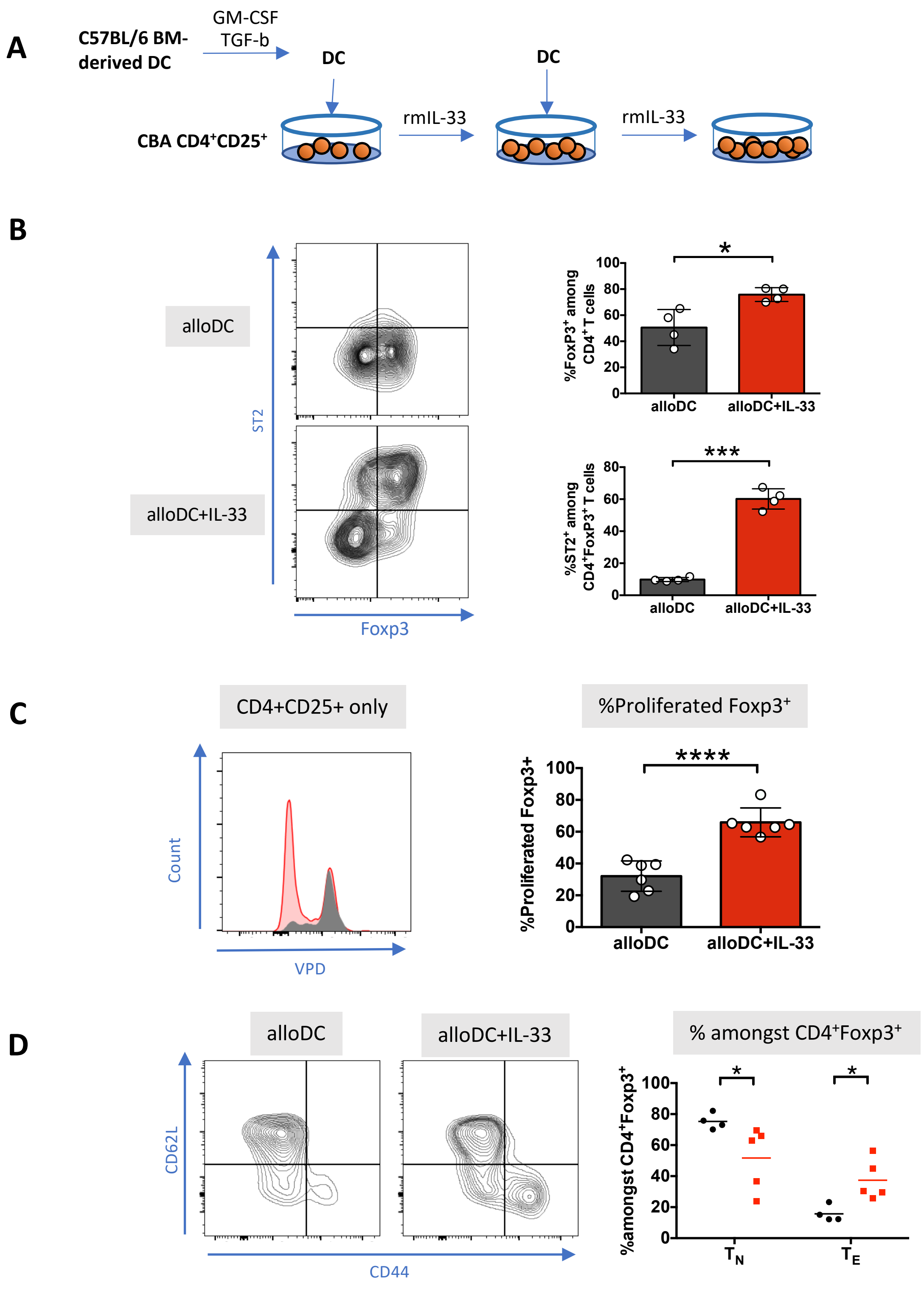

Supplementary Figure 2. (A) CD4+CD25+ Tregs were isolated from CBA/Ca (H-2k) spleens, stained with violet proliferation dye (VPD), and stimulated with/without IL-33 (10ng/mL) and C57BL/6 (H-2b) allogeneic DCs (GT-DC), which were generated in the presence of GM-CSF (10ng/mL) and TGF-β (2ng/mL). Cultures were restimulated with new GT-DC and IL-33 after one week and were harvested for flow cytometric analysis after two weeks. **(B)** Representative dot plots and graphs of expression of Foxp3 and ST2 of CD4+CD25+ cultures after stimulation with IL-33 and GT-DC. (unpaired *t* test, n=4) (**p*<0.05; ****p*<0.001; *****p*<0.0001; ns=not significant.) **(C)** Histogram and graph of Foxp3+ cells proliferation within CD4+CD25+ cultures. **(D)** Representative dot plots and graph of CD44 and CD62L expression within CD4+CD25+ cultures. (unpaired *t* test, n=4) (**p*<0.05; *****p*<0.0001; ns=not significant.

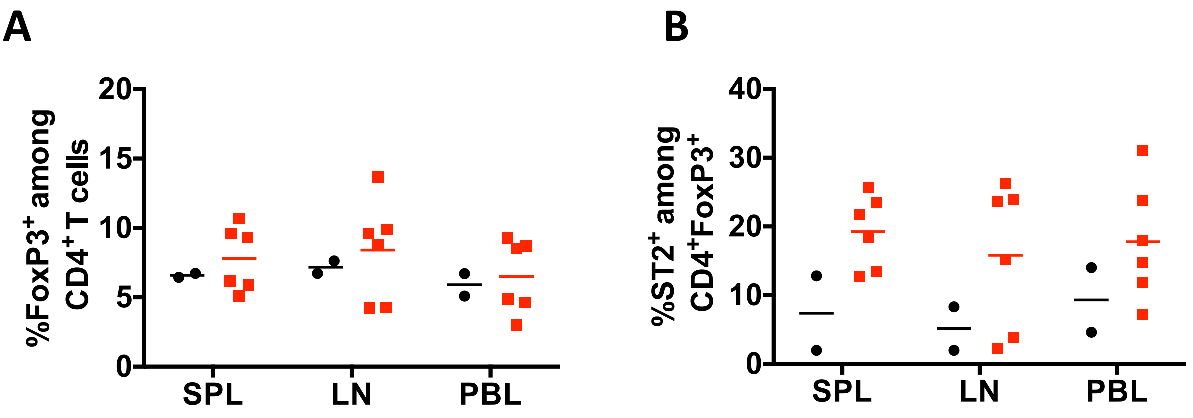

Supplementary Figure 3. CBA/Ca $Rag1^{-/-}$ ($H\text{-}2^k$) received control $CD4^+GFP^{neg}$ effector T cells (Teff) with or without $CD4^+GFP^+$ Tregs from $H\text{-}2^k$ Foxp3-GFP mice that were treated with PBS or IL-33 (1μg/day for 6 consecutive days and sacrificed on day 7). One day later, mice received an allogeneic fully-MHC mismatched $H\text{-}2^b$ skin allograft, which was monitored for rejection for 100 days post-transplantation. Mice that received Teffs and control (black, n=2) or IL-33-Tregs (red, n=6) and reached long-term graft survival (>100 days) were sacrificed at d100 and spleens (SPL), graft-draining lymph nodes (LN), and peripheral blood (PB) were harvested and analyzed by flow cytometry for Foxp3 **(A)** and ST2 **(B)** expression.

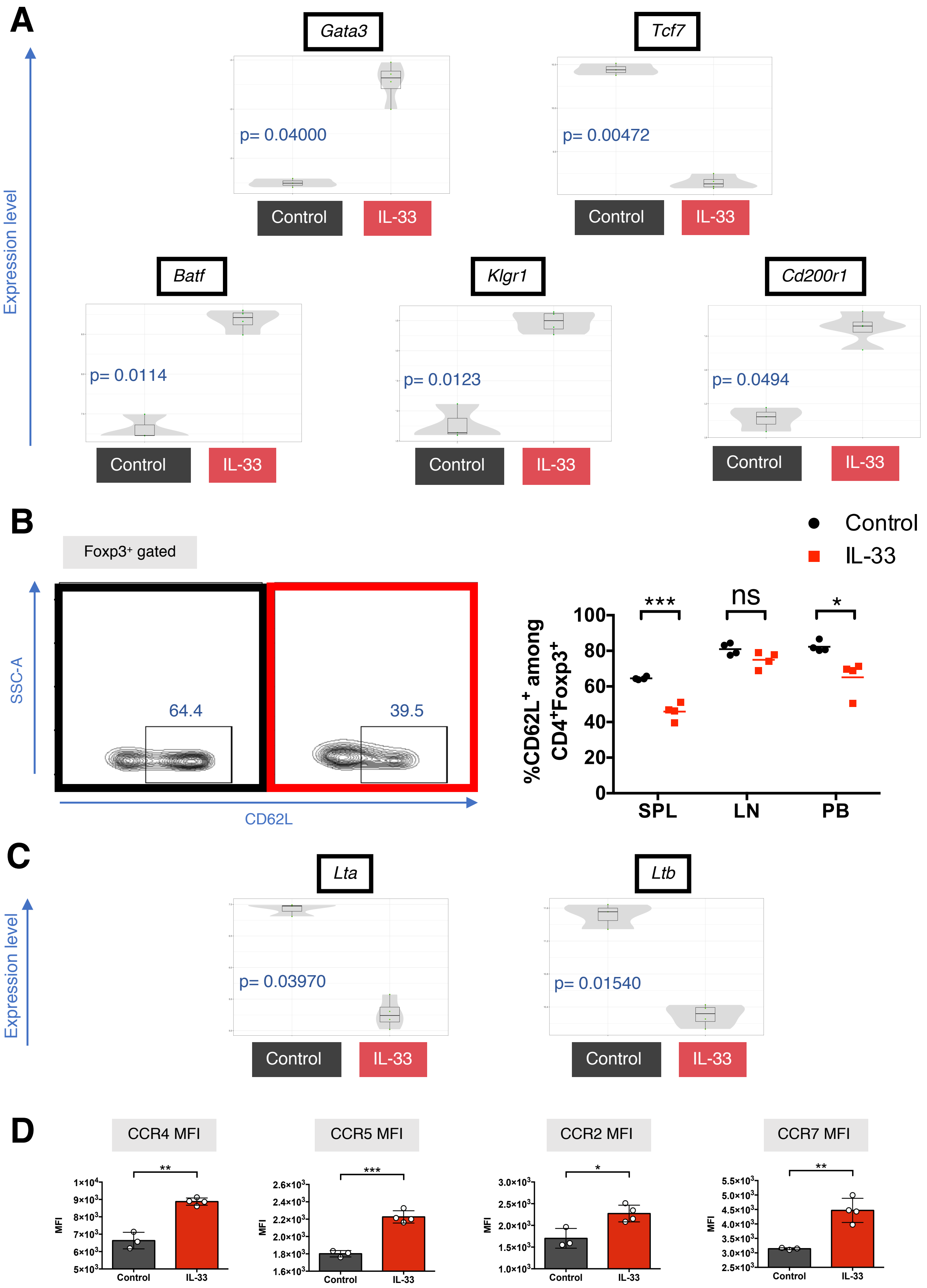

Supplementary Figure 4. (A and C) RNA from CBA/Ca (H-2k) splenocyte-derived CD4+CD25+ Tregs were FACS-sorted from PBS (n=3) or IL-33 treated H-2k mice (n=4, 1μg/day for 6 consecutive days and sacrificed on day 7) for gene expression analysis. **(A)** Genes *Tcf7*, *Gata3*, *Batf*, *Klrg1*, and *Cd200r1* are represented in univariate violin plots. **(B)** CBA/Ca (H-2k) mice were injected with either PBS (control, black) or recombinant IL-33 (red, 1μg/day for 6 consecutive days and sacrificed on day 7). Representative dotplots (from splenocytes) and graphs of expression of CD62L within CD4+Foxp3+ gated populations in the spleen (SPL), lymph node (LN), and peripheral blood (PB) (unpaired *t* test, n=4) (*$p<0.05$; ***$p<0.001$; ns=not significant). **(C)** Genes *Lta* and *Ltb* are represented in univariate violin plots. **(D)** Representative dotplots and graphs of showing MFI of select chemokine receptor molecules (unpaired *t* test, n=4) (*$p<0.05$; **$p<0.01$; ***$p<0.001$; ****$p<0.0001$; ns=not significant.)

A

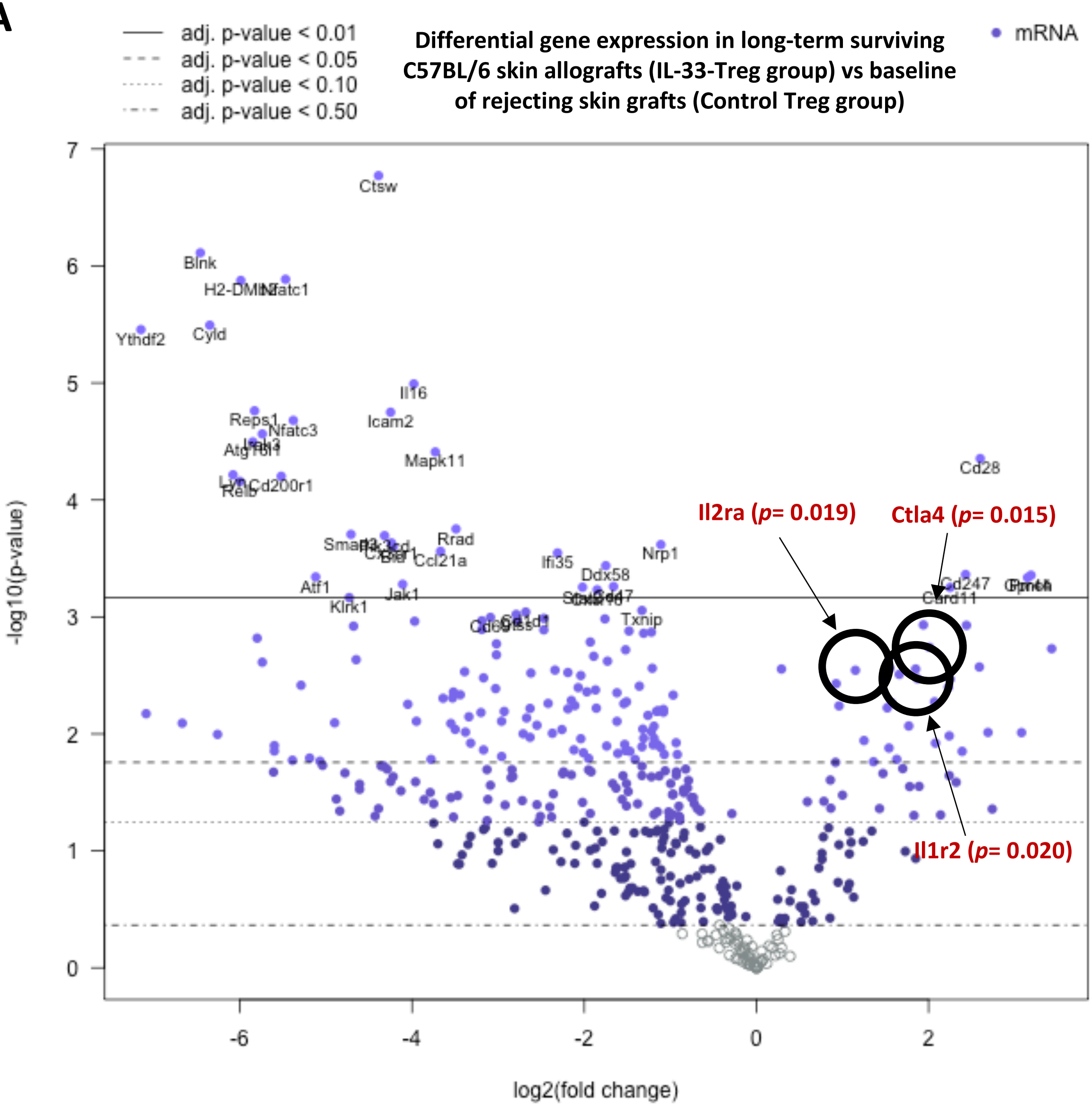

Supplementary Figure 5. CBA/Ca Rag1$^{-/-}$ (H-2^{k}) received control CD4^{+}GFP^{neg} effector T cells (Teff) with or without CD4^{+}GFP^{+} Tregs from H-2^{k} Foxp3-GFP mice that were treated with PBS or IL-33 (1μg/day for 6 consecutive days and sacrificed on day 7). One day later, mice received an allogeneic fully-MHC mismatched H-2^{b} skin allograft, which was monitored for rejection for 100 days post-transplantation. Rejecting skin grafts from the control Treg group (n=2) and grafts that reached long-term survival (>100 days) from the IL-33-Tregs group (n=2) were analyzed with Nanostring Transcriptomic analysis. Adjusted *p* value calculated with control of Benjamini-Hochberg False Discovery Rate (FDR) (Adjusted *p* value > 0.05 considered significant, FDR thresholds indicated within volcano plot.)
